# Supplementary material for: Zero-shot image classification based on class representation learning and attribute embedding learning
Source: PLoS One. 2025 Nov 17;20(11):e0332797. doi: 10.1371/journal.pone.0332797 (PMC12622808; doi:10.1371/journal.pone.0332797)
Supplement: Attachment — Submitted filename: response.pdf [file pone.0332797.s001.pdf]

Response to ‘PONE-D-25-22333’

We are very grateful to the anonymous reviewers and editors for their invaluable time and effort in reviewing our manuscript and particularly providing constructive comments and suggestions for significantly improving the manuscript. Following these comments and suggestions, we have made changes to the revised manuscript. A point-by-point reply to the reviewer’s comments is given below.

1. Response to Reviewer #1

Comment. 1: Figure 1 is missing from the main manuscript, which makes it difficult to fully understand the method’s pipeline.

Response: Thank you for your valuable and thoughtful comments. We sincerely apologize for the omission of Fig. 1 in the initial manuscript. This figure plays a crucial role in understanding the overall pipeline of the proposed method. In the revised version, we have added Fig. 1 to the main manuscript, which clearly illustrates the overall architecture and processing flow of our approach.

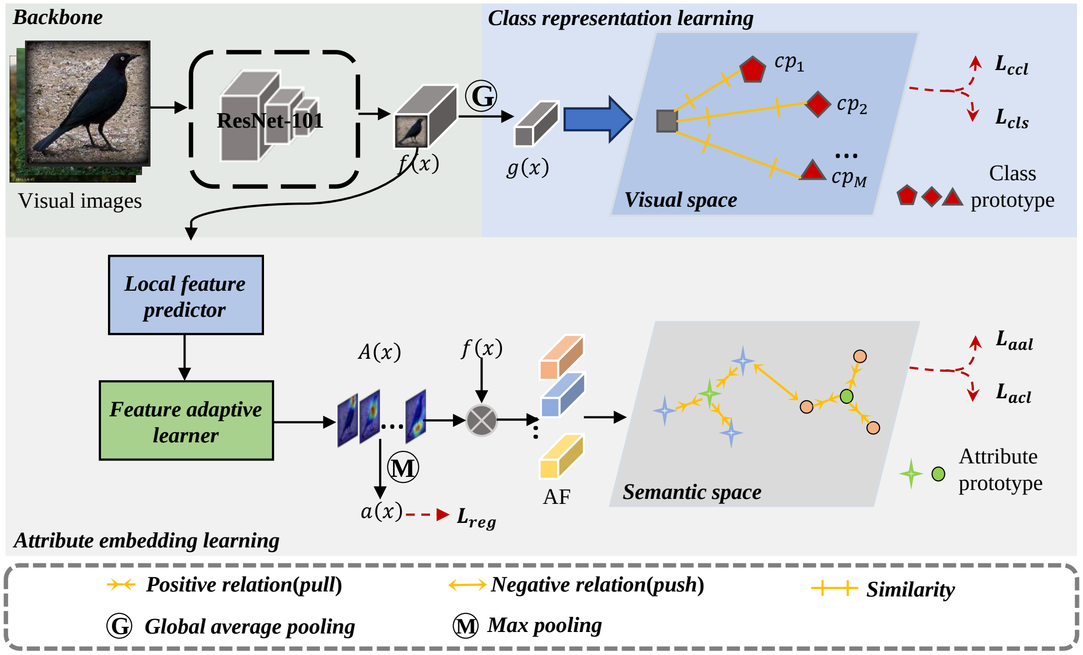

Figure 1: Illustration of our proposed framework, which contains two parts: class representation learning and attribute embedding learning. Class representation learning includes global feature alignment and class-level contrastive learning. Attribute embedding learning includes attribute localization, attribute feature extraction, attribute prototype alignment, and attribute-level contrastive learning.

Comment. 2: The attribute-level contrastive embedding bears strong similarity to prior work [1], but the paper does not clearly differentiate its approach or provide a comparative discussion. [1] Semantic Contrastive Embedding for Generalized Zero-Shot Learning. IJCV, 2022.

Response: Thank you for the valuable comment regarding the similarity between our attribute-level contrastive embedding and the work in [1]. We have carefully compared the design concepts and implementation details of both approaches and observed the following:

(1) The Semantic Contrastive Embedding in [1] primarily focuses on semantic-level contrastive learning to build a generalized attribute embedding space, emphasizing global alignment of semantic representations.

(2) In contrast, our method concentrates on attribute-level contrastive learning directly in the visual feature space, introducing a hard sample selection strategy that effectively filters out easy samples and strengthens the constraints on hard samples, thereby enhancing the discriminative power and robustness of attribute embeddings.

(3) Furthermore, we incorporate an adaptive softmax activation mechanism that dynamically normalizes attribute feature maps to significantly reduce noise interference, a component not addressed in [1].

To clarify these distinctions, we will add a dedicated section in the revised manuscript to discuss related work.

Comment. 3: Although the adaptive softmax activation is claimed as a key contribution, the paper lacks a dedicated section or sufficient analysis highlighting this component.

Response: We sincerely thank the reviewers for this constructive suggestion. The conventional softmax function applies a uniform normalization across all attribute channels, implicitly assuming similar importance and response distributions among attributes. However, in reality, the correlation and response strength between different attributes and local visual regions vary significantly. Uniform normalization may cause weakly responding attributes to be overwhelmed by noise, while strongly responding attributes might mask subtle differences, resulting in blurred attribute feature representations.

To address this, we introduce learnable scaling parameters $\rho_i \in [0, 1]$ for each attribute feature map, adjusting the activation values as follows:

$$A(x)_i = \text{Softmax}\big(A(x)_i \times \rho_i\big).$$

This can be interpreted as applying a dynamic "temperature" scaling to each attribute's response distribution. Smaller ρ_i values smooth the softmax outputs, suppressing noisy responses, whereas larger ρ_i values amplify discriminative attributes. This mechanism enables the model to adaptively optimize attribute feature quality, reducing irrelevant interference and enhancing the representational robustness of the attribute feature space. (**See the "Attribute Features Extraction" in our revised paper**).

Comment. 4: The baseline methods used for comparison are outdated. Including more recent state-of-the-art approaches would strengthen the experimental validation.

Response: Thanks for suggestions. To enhance the comprehensiveness of our comparative experiments, we have further included two recently proposed zero-shot classification methods: PFRN[2] (2025) and VENet-Res[3] (2025). These methods represent the latest advancements in the field and are highly representative of current state-of-the-art techniques. Our proposed CRAE framework consistently outperforms both methods across multiple benchmark datasets, demonstrating its robustness and competitiveness.(**See the "Tab. 3" in our revised paper**)

[2] Hu L, Cao W, Zhang Z, et al. Progressive Feature Reconstruction Network for Zero-shot Learning[J]. IEEE Transactions on Circuits and Systems for Video Technology, 2025.

[3] Ge J, Liu Z, Li P, et al. Denoised and Dynamic Alignment Enhancement for Zero-Shot Learning[J]. IEEE Transactions on Image Processing, 2025.

2. Response to Reviewer #2

Comment. 1: While the combination of adaptive softmax and dual contrastive learning is compelling, a more in-depth theoretical explanation or deep analysis would be also necessary.

Response: Thank you for your very constructive advice. We provide the following detailed analysis to clarify the underlying principles and justify their effectiveness:

1. Theoretical Basis of Adaptive Softmax Activation The conventional softmax function applies a uniform normalization across all attribute channels, implicitly assuming similar importance and response distributions among attributes. However, in reality, the correlation and response strength between different attributes and local visual regions vary significantly. Uniform normalization may cause weakly responding attributes to be overwhelmed by noise, while strongly responding attributes might mask subtle differences, resulting in blurred attribute feature representations.

To address this, we introduce learnable scaling parameters $\rho_i \in [0, 1]$ for each attribute feature map, adjusting the activation values as follows:

$$A(x)_i = \text{Softmax}\big(A(x)_i \times \rho_i\big).$$

This can be interpreted as applying a dynamic "temperature" scaling to each attribute's response distribution. Smaller ρ_i values smooth the softmax outputs, suppressing noisy responses, whereas larger ρ_i values amplify discriminative attributes. This mechanism enables the model to adaptively optimize attribute feature quality, reducing irrelevant interference and enhancing the representational robustness of the attribute feature space.

2. Synergistic Mechanism and Advantages of Dual Contrastive Learning Our framework integrates two complementary contrastive learning components:

- **Class-level contrastive learning:** By maximizing similarity among global features of samples within the same class and minimizing similarity between different classes, it strengthens coarse-grained category discrimination boundaries, improving the model's ability to differentiate visually similar categories.
- **Attribute-level contrastive learning:** At a finer granularity, it utilizes hard positive and negative mining strategies to focus on challenging attribute feature pairs, reducing intra-attribute variation across samples and minimizing inter-attribute confusion. This effectively refines decision boundaries within the attribute embedding space.

Together, these components enable the model to achieve both global class discrimination and fine-grained attribute distinction, substantially enhancing the discriminative power and generalization ability of the learned visual representations.(**See the "Class-level Contrastive Learning" and "Attribute-level Contrastive Learning" in our revised paper**)

Comment. 2: The choice of hard positive/negative thresholds (and) is important but appears empirically chosen. It should discuss the rationale and potential to generalize or learn these thresholds adaptively.

Response: We sincerely thank the reviewer for this advice. We thank the reviewer for highlighting the importance of the hard sample selection thresholds μ and ε. We fully agree that their proper choice significantly influences the contrastive learning process and overall model performance. In our study, we conducted a systematic ablation analysis on μ and ε using the validation sets of the CUB and SUN datasets (as shown in Fig. 2). The results demonstrate that the best performance under both conventional and generalized zero-shot learning settings is achieved when $\mu = 0.3$ and $\varepsilon = 0.5$. Notably, the performance degrades when these values are too small (e.g., 0.1) or too large (e.g., 0.5 for μ on CUB), suggesting that both under- and over-filtering can harm the learning process by either preserving too many trivial samples or discarding too many training instances.

In the revised manuscript, we have also included a discussion on the potential for generalization and adaptive learning of these thresholds. In future work, adaptive strategies could be explored—such as using percentile-based statistics on similarity distributions to dynamically set μ and ε, or incorporating learnable threshold generation modules that adjust based on training dynamics. These enhancements would improve the robustness and adaptability of our framework in more complex zero-shot scenarios. (**See "The Importance of Hard Samples Selection" in our revised paper**)

Comment. 3: The ablation study would benefit from more visual evidence (e.g., t-SNE plots) comparing feature distributions.

Response: Thank you for your valuable suggestion. We fully agree that incorporating visual evidence into the ablation study can provide more intuitive insights into the effect of each module on the feature distributions, thereby enhancing the persuasiveness of the analysis. Accordingly, in the revised manuscript, we have added visualizations (see Fig. 3, Fig. 4 and Fig. 5) that illustrate the distribution of extracted features under different module configurations using t-SNE plots. We believe these visualizations further validate the effectiveness of our proposed method in enhancing feature separability and discriminative ability.(**See the "Qualitative Results" in our revised paper**).

t-SNE Visualizations of Class Representations

As shown in Fig. 3, t-SNE visualization is employed to illustrate the class representations (global visual features $g(x)$) of seen and unseen classes on the test datasets of CUB and AWA2. For experimentation, 10 seen classes and 10 unseen classes are randomly selected from the CUB dataset, along with the top 10 seen classes and all unseen classes from the AWA2 dataset. Different colors represent different classes. The visualization results reveal that, for both datasets, the global visual features of the same class tend to cluster together, indicating good intra-class consistency, while the features of different classes are dispersed, indicating distinct inter-class differences. This demonstrates that the CRAE model can learn highly discriminative visual embedding spaces, which is advantageous for image classification tasks, as the class-level contrastive learning constrains the global visual features. Additionally, the visualization results of unseen classes on both datasets demonstrate the model's proficiency in distinguishing unseen class images. This is attributed to the attribute embedding learning branch assisting the class representation learning branch, enriching the global visual features with deeper semantic information.

t-SNE Visualizations of Attribute Features

To assess the discriminative nature of attribute embedding space, 15 attributes were randomly selected from each of the three datasets, resulting in a total of 2048 attribute-level features. These features were then

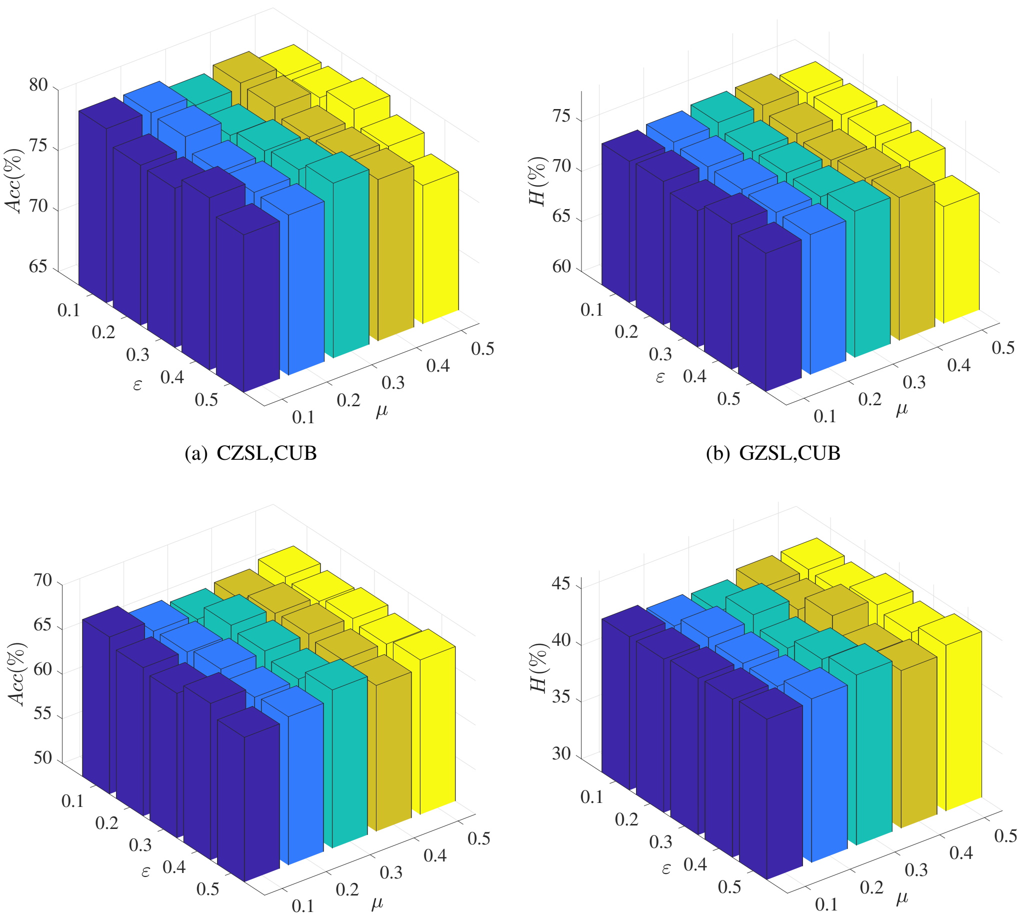

(a) CZSL,CUB (b) GZSL,CUB

(c) CZSL,SUN (d) GZSL,SUN

Figure 2: The classification results on CUB and SUN with different values of μ and ε.

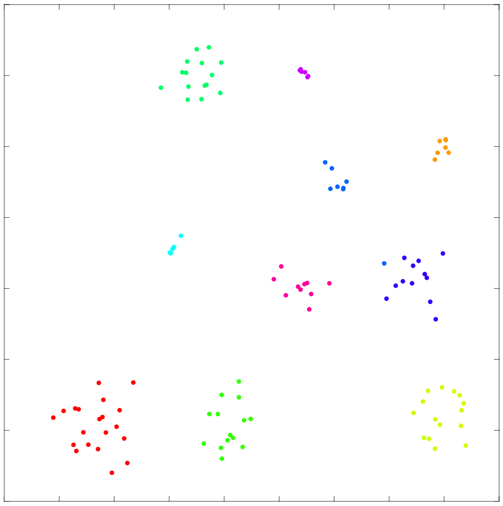

(a) CUB,seen classes

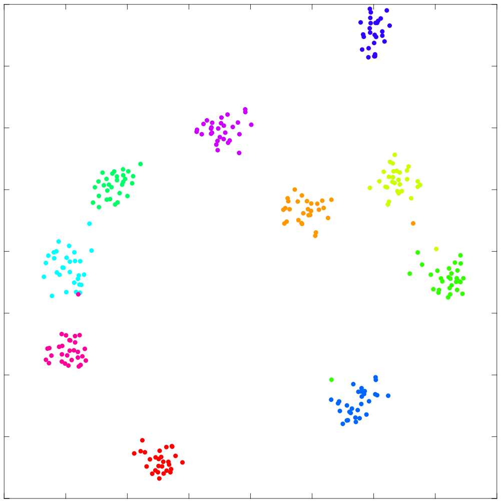

(b) CUB,unseen classes

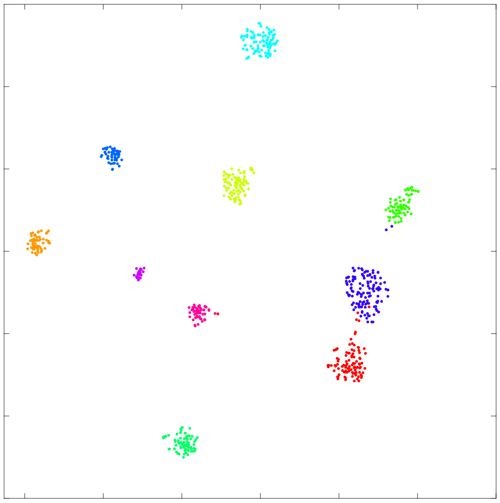

(c) AWA2,seen classes

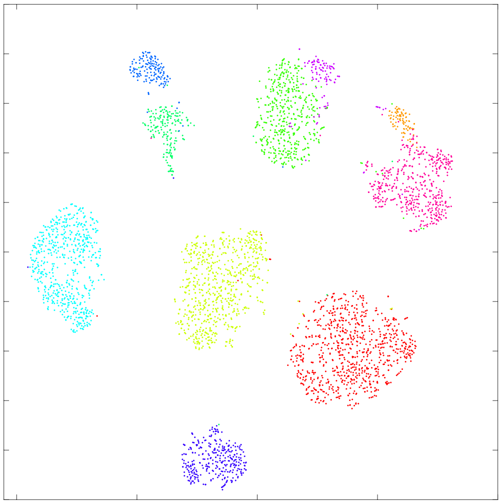

(d) AWA2,unseen classes

Figure 3: The t-SNE visualization of class representations for seen and unseen classes on CUB and AWA2.

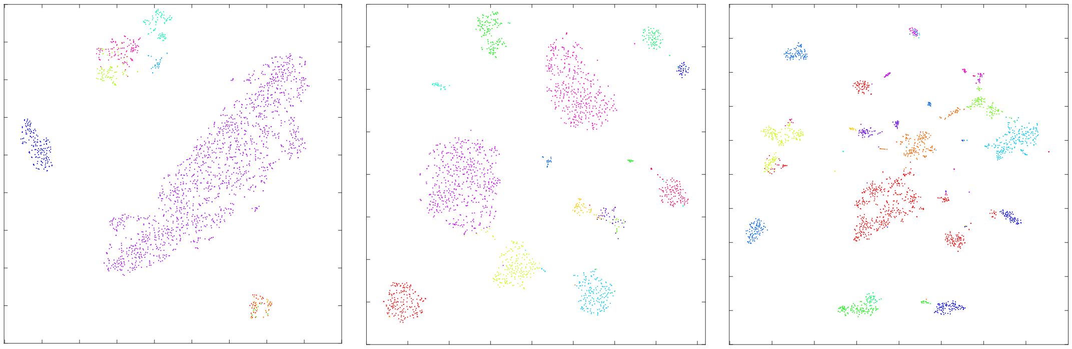

(a) CUB (b) SUN (c) AWA2

Figure 4: The t-SNE visualization of attribute features on CUB, SUN, and AWA2.

visualized using t-SNE, as shown in Fig. 4. Features of the same color share the same attribute label, while features of different colors have different attribute labels. It can be observed that the attribute features of different attributes are easily separated in the attribute embedding space, with clear boundaries between them. Conversely, features of the same attribute tend to cluster together, indicating good intra-class consistency. This confirms that the CRAE model can reduce the differences between the same attribute in the embedding space and has the ability to accurately identify attributes, which helps overcome domain shift issues. This is primarily because the attribute-level contrastive loss based on hard sample selection can constrain the distribution of attribute-level features in the attribute embedding space, enhancing the discriminative nature of attribute-level features. Further observation of Fig. 4 reveals that some attributes (e.g., mouth attribute represented by red dots in Fig. 4(3)) have more feature points, while some attributes (e.g., hoof attribute represented by green dots in Fig. 4(3)) have fewer feature points, reflecting the different frequencies of occurrence of different attributes in the dataset. Moreover, Fig. 4 also reflects the semantic similarity between different attributes; for example, the semantic similarity between tail and hoof is less than that between claw and hoof.

Comment. 4: May, it would be better to give a brief discussion on potential challenges (e.g., missing attributes, noisy labels, class imbalances) and future directions.

Response: We thank the reviewer for the thoughtful suggestion. We agree that a brief discussion on potential challenges and future research directions would enhance the completeness of the work and provide a broader perspective to the readers. Accordingly, we have added the following paragraph to the Conclusion section of the revised manuscript: Despite the strong performance of CRAE, several challenges remain for future exploration. First, the model's performance may be affected by missing or inaccurate attribute annotations, which can lead to misaligned feature representations. Second, the presence of noisy labels in training data, especially for fine-grained categories, may degrade the robustness of contrastive learning. Third, class imbalance between seen and unseen categories remains an inherent challenge in zero-shot settings. Future research may explore robust attribute completion techniques, noise-resilient learning strategies, and meta-learning-based adaptation to further improve the generalization and applicability of zero-shot learning methods.(**See the "Conclusion section" in our revised paper**)

Comment. 5: Some equations (e.g., Eq. 5) are complex and could be reformatted for readability.

Response: Thanks so much for the careful reading. In the revised manuscript, we have reformatted and

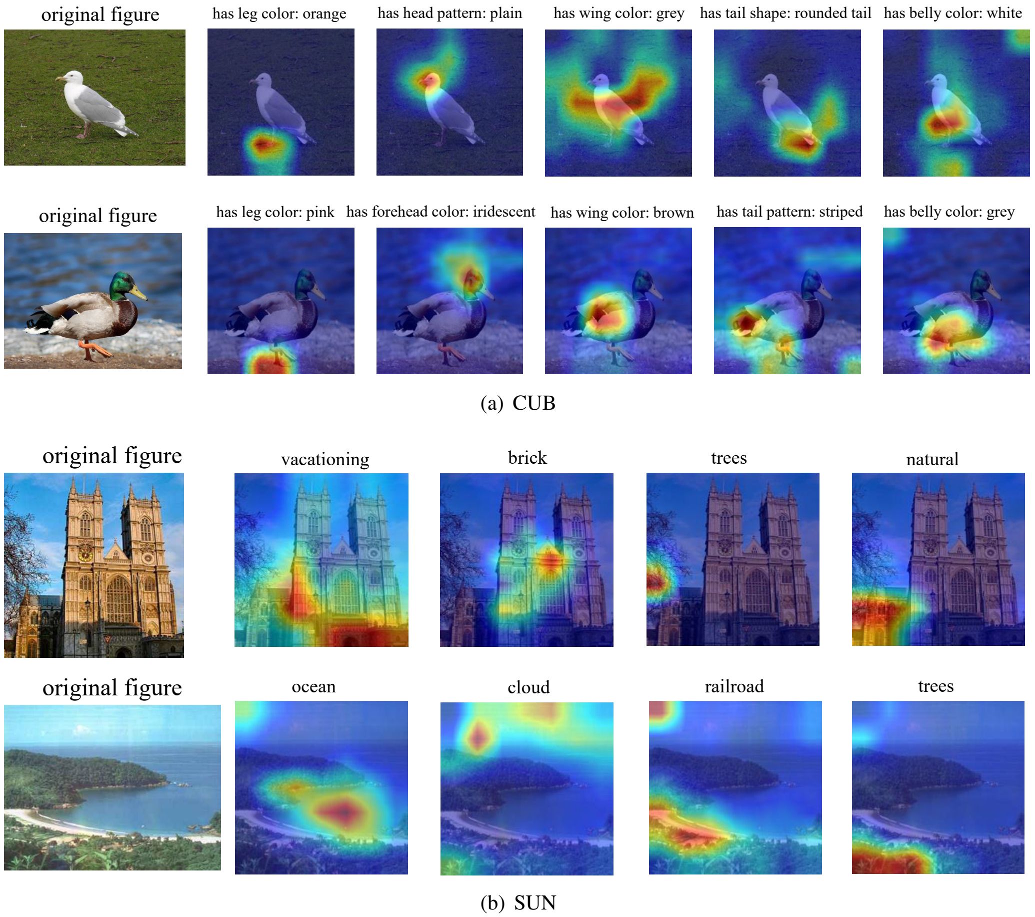

Figure 5: Visualization results of attribute feature maps on CUB and SUN datasets.

refined Equations (4) and (5) to make the expressions more concise and clear, thereby improving their readability and interpretability. (**See the Equations (4) and (5) in our revised paper**).
